# Nutritional Status and Its Determinants among Adult Cancer Patients Undergoing Chemotherapy Treatment at Hawassa University Comprehensive Specialized Hospital, Hawassa, Southern Ethiopia

**DOI:** 10.1155/2022/8740272

**Published:** 2022-09-28

**Authors:** Ahmed Nuru Muhamed, Berihun Bantie, Endalk Getasew Hiruy, Sahlu Mitku Shiferaw, Dessie Temesgen Aycheh, Melsew Dagne Abate

**Affiliations:** ^1^Department of Nursing, College of Medicine and Health Sciences, Wolkite University, Wolkite, SNNPR, Ethiopia; ^2^Department of Adult Health Nursing, College of Health Sciences, Debre Tabor University, Debre Tabor, Ethiopia; ^3^St. Peter Specialized Hospital, Addis Ababa, Ethiopia; ^4^Debre Tabor Health Sciences College, Department of Nursing, Debre Tabor, Ethiopia; ^5^Department of Adult Health Nursing, College of Health Sciences, Woldia University, Woldia, Ethiopia

## Abstract

**Background:**

Malnutrition is a common problem in cancer patients. It has an impact on all aspects of the patient's life such as increasing the risk of infection, treatment toxicity, hospital stay, and health-care costs. Factors influencing the nutritional status of adult cancer patients undertaking chemotherapy treatment in Ethiopia have not been thoroughly investigated. As a result, the purpose of this study is to assess the nutritional status and its determinants among adult cancer patients undergoing chemotherapy treatment at Hawassa University Comprehensive Specialized Hospital.

**Objectives:**

The objective of this study is to determine the nutritional status and its determinants among adult cancer patients undergoing chemotherapy treatment at Hawassa University Comprehensive Specialized Hospital.

**Methods:**

A cross-sectional study was conducted among adult cancer patients undergoing chemotherapy treatment at Hawassa University Comprehensive Specialized Hospital Oncology Treatment Center, from January to May 2021. The data were gathered through a face-to-face interview and chart review method. Epi Data 4.6 was used to enter the data, which was then exported to SPSS version 25 for statistical analysis. Multivariable logistic regression analysis was used to determine the association between nutritional status and potential risk factors. A *P* value less than 0.05 was used to determine statistical significance.

**Result:**

This study revealed that 48.1% of participants have some level of malnutrition. Lowest wealth index AOR 0.06 (0.016–0.2), food insecurity AOR 0.1 (0.05–0.24), vomiting AOR 0.2 (0.110–.444), poor appetite AOR 0.2 (0.11–0.44), no diarrhea AOR 2.6 (1.34–5.00), and poor functioning AOR 0.3 (0.2–0.54) were significantly associated with good nutritional status. *Conclusion and Recommendation*. The prevalence of malnutrition among adult cancer patients undergoing chemotherapy treatment at HUCSH was high. Wealth index, food security, poor appetite, diarrhea, and performance status were significantly correlated with the nutritional status of the patients. To improve the patient's nutritional status, economic support, early nutritional screening, and assessment, management of chemotherapy-induced symptoms should be considered.

## 1. Introduction

Cancer is a disease in which abnormal cells develop without control, these cells develop and invade other cells and spread to different sites in the body causing disease and, if left untreated, could lead to the death of an individual [1]. Cancer is the world's second leading cause of death [2]. Globally, there were 17.0 million new cancer cases and 9.6 million deaths from cancer in 2018 [2]. About 70% of cancer-related deaths were reported in low- and middle-income countries [3]. In Ethiopia, cancer is responsible for 77,352 new cases and 51,865 cancer deaths in 2020 [4].

Cancer patients are vulnerable to nutritional deficiency as a result of the combined effects of the disease and its treatment [5]. Nutrition has a positive role in improving patients' overall health. On the other hand, malnutrition affects all aspects of a patient's life by increasing the risk of infection, delayed wound healing, increasing treatment toxicity, extending hospital stay, and increasing health-care costs [6].

Malnutrition is defined as an acute or chronic nutritional condition characterized by nutrient excess or deficiency, energy imbalance, and inflammatory activity that results in a change in body composition, impairment of function, and clinical outcome [6].

Cancer and its treatment have an impact on the nutritional status by altering the metabolic system, altered food taste, and reducing food intake [7]. It causes changes in physiological and psychological functions [7], which may have an impact on quality of life [7]. Indeed, acute and chronic symptoms associated with antineoplastic treatments frequently have a negative impact on the nutritional status of the patients [8–10].

Chemotherapy has a number of side effects, including nausea, vomiting, anorexia, diarrhea, and constipation, all of have an effect on the patient's life [6]. Patients are more likely to become malnourished in these circumstances, especially if chemotherapy treatment is given more often and for long periods [6, 11].

Even though cancer and its treatment raise the risk of malnutrition, early assessment of nutritional status is crucial for early nutritional management and thus increasing the patient's survival [10]. Despite the high prevalence of malnutrition in cancer patients, only a few studies have examined the nutritional condition of adult cancer patients in Ethiopia, with all previous studies took place in Addis Ababa (the capital city of Ethiopia). Furthermore, there are key issues that affect cancer patients' nutritional health that have yet to be addressed, particularly in our country's setting, such as length of sickness [10], food security [12], anxiety [13, 14], and depression [13, 15].

This study aims to examine the nutritional status of adult cancer patients who are undergoing chemotherapy treatment at Hawassa University Comprehensive Specialized Hospital (HUCSH). As a result, this research will help researchers to better understand the incidence of malnutrition and the factors that contribute to it. It will also aid healthcare workers to emphasize the prevention of malnutrition. As a result, early detection and management of malnutrition will reduce mortality, morbidity, and length of stay in the hospital, improve healing, and reduce costs.

## 2. Methodology

### 2.1. Study Area and Period

The study was conducted at the Cancer Treatment Center of HUCSH from January to May 2021. Hawassa University Comprehensive Specialized Hospital is one of the teaching and referral hospitals providing cancer treatment for the southern nation's nationalities peoples and Sidama National Regional State of Ethiopia and is under Hawassa University.

Hawassa University Comprehensive Specialized Hospital is located in Hawassa city which is 275 km far from Addis Ababa. The oncology unit of the hospital provides diagnostic, surgical, and chemotherapy treatment services. The Oncology Treatment Center contains a total of 12 beds for inpatient treatment, and the treatment was given in three days per week.

### 2.2. Study Design

An institutional-based cross-sectional study design was conducted.

### 2.3. Population

#### 2.3.1. Source Population

The source populations were all adult cancer patients who are on chemotherapy treatment follow-up at the HUCSH Cancer Treatment Center.

#### 2.3.2. Study Population

The study populations were all adult cancer patients undertaking chemotherapy treatment during the data collection period at HUCSH.

### 2.4. Inclusion and Exclusion Criteria

Inclusion Criteria Patients were included in the study if they were 18 years or older, had no hospitalization in the month before the study (except for routine chemotherapy), and had received at least one cycle of chemotherapy treatment, regardless of the site of cancer.

#### 2.4.1. Exclusion Criteria

The exclusion criteria include the presence of active illness, hearing, or communication impairment.

### 2.5. Study Variables

#### 2.5.1. Dependent Variable

It is nutritional status.

#### 2.5.2. Independent Variables

*(1) Socio-demographic factors*. age, sex, educational status, marital status, residence, religion, occupation, wealth index, and food security.

*(2) Life Style Factors*. They are consumption of alcohol, cigarette smoking, and chat chewing.

*(3) Clinical Information*. It includes type of cancer, duration of illness, number of chemotherapy cycles, stage of cancer, performance status, comorbidities, anxiety, and depression.

*(4) Symptoms*. They are altered taste, poor appetite, dry mouth, pain, fatigue, mouth ulcer, nausea, vomiting, diarrhea, and constipation.

### 2.6. Operational Definition

1. Subjective global assessment score of nutritional status: according to the sum score of points assigned to each item, patients were classified as well nourished: <17 points, and malnourished (moderate and severe): ≥17 points [14].
2. Performance status: this is the assessment of the level of function and ability of self-care using the World Health Organization Performance Status measuring scale ranging from 0 (fully active) to 4 (bedridden) [16].
3. Food insecurity is a state that exists when people do not have adequate physical, social, or economic access to sufficient, safe, and nutritious food to meet their dietary needs for an active and healthy lifestyle [17].
4. The Household Food Insecurity Access Scale is a set of questions about food access that are used to distinguish food-secure from food-insecure households in various cultural contexts [18].

### 2.7. Sample Size Determination

All adult cancer patients who visited the HUCSH Cancer Treatment Center for chemotherapy treatment during the data collection period were included consequently. The sample size was calculated using a single population proportion formula with a 5% margin of error (*d*) and a 95% confidence interval (alpha = 0.05), and the proportion of malnutrition in cancer patients in Ethiopia at TASH was 27.5 percent (*P*=0.275) [17].

(1) $$\begin{array}{c} ni=Z^{2}\frac{P\left(1-P\right)}{d^{2}}, \end{array}$$

where ni = initial sample size. Z*α*/2 = 1.96 (*Z* = score corresponds to 95% confidence level). *P* = proportion of affected quality of life of cancer patients. q = proportion of not affected quality of life of cancer patients. d2 = margin of error (0.05)

(2) $$\begin{array}{c} ni=1.96^{2}\frac{0.275\left(0.725\right)}{0.05^{2}}. \end{array}$$

Considering 10% of contingency for nonresponses, the final sample size will be 338.

### 2.8. Data Collection and Quality Control

There were six parts to the data collection: initially, after evaluating the literature, the authors constructed questionnaires to collect socio-demographic data, and clinical information about the disorders and treatments was collected from the medical chart.

Second, a wealth index questionnaire consists of 21 questions that are used to measure housing conditions and household characteristics. Principal component analysis was used to compute and minimize the number of variables (PCA). Then, using an Ethiopian demographic health survey, they were divided into five quintiles [19].

To assess the patient's nutritional status, the patient-generated subjective global assessment (PG-SGA) tool was used. This tool consists of a patient history emphasizing weight loss, gastrointestinal symptoms such as nausea and vomiting, and a physical examination emphasizing subcutaneous fat tissue loss and muscle wasting [20].

Fourth, the Household Food Insecurity Access Scale (HFIAS) was used to assess food insecurity in households.

It consists of nine questions about food insecurity, with response options of never, rarely, sometimes, or often. The highest HFIAS score is 27, and the higher the score, the greater the food insecurity [15].

Fifth, the World Health Organization Performance Status (WHO-PS) questionnaire was used to assess the patient's performance status.

It is frequently used to assess how the condition affects the patient's ability to perform daily duties, which are graded on a scale of 0 (completely active) to 4 (bedridden), with a score of 0–1 indicating good status and 2–4 indicating poor status [16].

Sixth, the hospital anxiety and depression questionnaire was used to assess the patient's anxiety and depression levels. It is a valid and reliable questionnaire used to assess anxiety and depression in the general population [21]. The HAD consists of 14 items divided into two subscales (anxiety and depression), each with seven items, and is scored on a four-point Likert scale ranging from 0 to 3, with 0 being the most pleasant response and 3 being the least pleasant. Each subscale's score is computed, and values of 11 or higher are considered to indicate depression or anxiety disorder. Scores of 7 or less indicate that the person should not be considered a case, while scores of 8 to 10 indicate that the findings are questionable [21, 22].

### 2.9. Data Analysis

Data were entered into Epi Data 4.2 software, cleaned and coded, and then exported to SPSS version 25 for analysis (IBM SPSS, Chicago, IL, USA). SPSS Inc. was a software house headquartered in Chicago, USA. It is a foremost global manufacturer of software used in data analysis, data management, reporting, and modeling [23]. Categorical variables were expressed using descriptive statistics (frequency and percentages) and continuous variables using mean and standard deviation. Bivariable analyses were used to investigate the first connection between each independent variable and the dependent variable. Then, to account for cofounders and identify the predictors of nutritional status, those independent variables with a *P* value of less than 0.25 were entered into multivariable logistic regression. The statistical significance was determined by a *P* value of less than 0.05, and the strength of the association was determined by an OR with a 95 percent confidence interval. The Hosmer and Lemeshow goodness of fit test (*P* value = 0.223) was used to assess model fitness.

## 3. Results and Discussion

### 3.1. Socio-Demographic Characteristics of the Respondents

Out of 338 samples, 324 participated in the study having a response rate of 95.86%, 223 (68.8%) were female, and 175 (57.8%) were below the age group of 50 with a mean (SD) age of 45.81 years (10.98). More than half of the participants, 66 (54.2%), have no formal education. About 231 (71.3%) were married, and 215 (66.4%) of the patients were rural residents. Regarding occupation, 125 (37.7%) were housewives and 85 (27.4) were farmers. More than half of the study participants 53.3% have a lowest and second wealth index quintal (Table 1).

### 3.2. Lifestyle of Respondents

**Figure 1:**
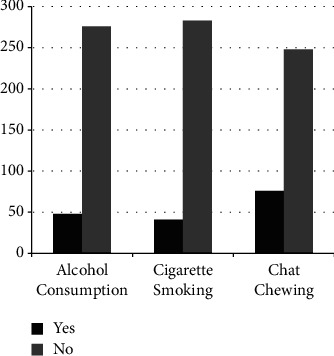
The lifestyle of adult cancer patients undertaking chemotherapy at HUCSH, Hawassa, Southern Ethiopia, 2021.

48 (14.8%), 42 (12.9%), and 76(23.4%) of the respondents had history of alcohol consumption, cigarette smoking, and chat chewing, respectively (Figure 1).

### 3.3. Clinical Characteristics of the Participants

Breast cancer was the leading type of cancer accounting for 25.5% of the respondents, followed by GI cancer 23.9%. The mean length of time since their diagnosis of cancer was 14.95 months. One hundred and thirty two (42.3%) of the participants were in stage III of cancer. Concerning the current chemotherapy treatment, 89 (28.7%) participants were in their fourth cycle of treatment. Seventy one (21.9%) of the participants had comorbid diseases, among these 54.9% of the participants had hypertension. The mean (SD) of anxiety and depression subscale scores was 8.83 (3.287) and 9.29 (4.177), respectively (Table 2).

### 3.4. Nutritional Status of Cancer Patients

One hundred and sixty eight (51.9%) of the participants are well nourished, and 156 (48.1%) are malnourished (moderate and severe malnutrition) (Table 3).

### 3.5. Symptoms of Cancer Patients

**Figure 2:**
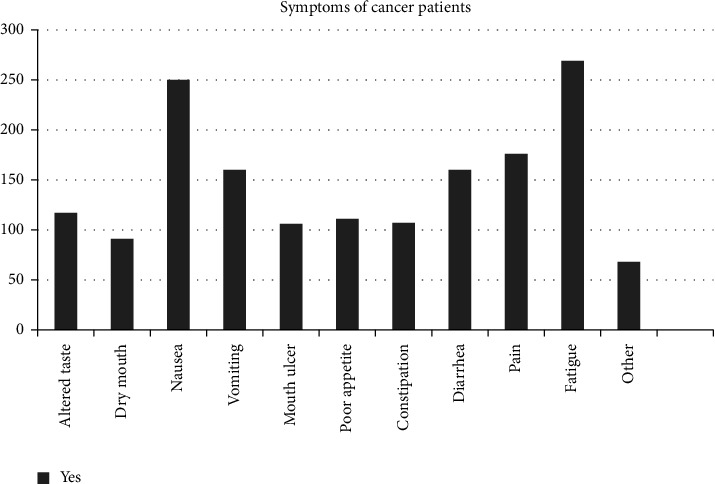
Symptoms of adult cancer patients undertaking chemotherapy treatment at HUCSH, Hawassa, Southern Ethiopia.

The common symptoms that appeared among cancer patients were fatigue 269 (83%), nausea 250 (77.2%), appetite loss 111 (34.3%), diarrhea 160 (49.4%), and vomiting 106 (32.7%) (Figure 2).

### 3.6. Determinants of Nutritional Status

In this study, multiple factors were associated with nutritional status in adult cancer patients. The bivariable logistic regression analysis shows that educational status, wealth index, food security, type of cancer, stage of cancer, the cycle of chemotherapy, the presence of comorbidities, anxiety, performance status, nausea, poor appetite, vomiting, and diarrhea were significantly correlated with nutritional status at *P* < 0.25 and entered into the multivariable analysis.

In the multivariable analysis, wealth index, food security, poor appetite, vomiting, diarrhea, and performance status had a significant association with nutritional status. The multivariable analysis showed that adult cancer patients with vomiting were 80% less likely to have good nutritional status, AOR 0.2 (0.110–444).

Patients that have the lowest wealth index were 94% less likely to be well nourished AOR 0.06 (0.016–0.2) compared to patients with the highest wealth index. Patients with food insecurity were 90% less likely to be well nourished AOR 0.1 (0.05–0.24) than patients with secured food. Similarly, patients who have poor appetite are 80% less likely to be well nourished than patients without appetite loss with AOR 0.2 (0.11–0.44). Those patients that have no diarrhea were 2.6 times more likely to have normal nutritional status than patients with diarrhea with AOR 2.6 (1.34–5.00). Patients that have poor functioning are 60% less likely to be well nourished than patients with good performance status with AOR 0.3 (0.2–0.54) (Table 4).

## 4. Discussion

Cancer patients are vulnerable to nutritional deficiency as a result of the combined effects of the disease and its treatment. Nutritional deficiency has an impact on all spheres of the patient's life by increasing the risk of infection, increasing treatment toxicity, delaying wound healing, prolonging hospital stay, and increasing health-related costs. In Ethiopia, nutritional assessment is not performed routinely on cancer patients. An institution-based cross-sectional study was aimed to assess the nutritional status and associated factors among adult cancer patients undergoing chemotherapy treatment at Hawassa University Comprehensive Specialized Hospital, Ethiopia, 2021.

This study has shown that the overall prevalence of malnutrition among adult cancer patients receiving chemotherapy was 48.1%. This finding was in line with studies done in Malaysia (43.5%) [24], Oman (40.21%) [25], and Ethiopia at Addis Ababa (47.1%) [26]. However, the finding of this study was higher than the study done in New Zealand (32%) [26], Vietnaam (34.1%) [27], Iran (6%) [5], and Japan (19%) [28]. A high level of malnutrition in this study could be explained by low socioeconomic status, differences in the assessment method used, less resourced health facilities, and lack of integration of dieticians in the treatment protocol of cancer [13, 14].

The nutritional status found in this study was associated with the economic conditions of the patients, patient with the lowest economic status was 94 times less likely to be well nurished than patients with the highest economic status, AOR 0.06 (0.02–0.2). This finding was in line with studies in the USA, Vietnam, and Kenya [25, 27, 29]. This can be explained by patients with a good financial status that can access a diversified nutritional diet and special supportive treatments that can promote the health status of the patient.

In this study, patients with insecure food were 90% less likely to be well nourished than patients with secured food with AOR 0.1 (0.05–0.24). This finding was in line with studies in Iran, Malaysia, Saudi Arabia, and Kenya [6, 12, 23, 30]. This can be explained by food insecurity in households leading to a change in dietary practices and lack of adequate food that can lead to poor nutritional status, so this can increase the burden of symptoms, the length of hospital stay, and poor prognosis [29].

Appetite loss and diarrhea are significantly associated with the nutritional status of adult cancer patients. This finding was consistent with studies reported from Libya, [31] India, [32] and Korea [33]. This may be due to cancer therapy affecting the nutritional status of the patient through altering the metabolic system and changes in food tests, resulting in damage to normal tissues [33].

In this study, a significant association was found between performance statuses with the nutritional status of participants. Functional performance activity is a factor that deserves attention, considering that individuals with limited functional capacity have difficulties in the preparation of food, and basic life care [16]. The results of this study support this statement because it was observed that patients with poor functional status were 60% less likely to have normal nutritional status than mobile patients. This finding was in line with other studies [5,14]. Poor functional status is a known factor unfavorably affecting the physical, psychological, and social health of the patients [16].

The potential limitation of this study was that food insecurity is assessed based on a one-month recall of occurrence, and social desirability bias may increase or decrease. Some variables like dietary diversity, family size of the patients, and treatment adherences were not determined which could be considered as a limitation. This study address anxiety and depression in relation to nutritional status.

## 5. Conclussion

The prevalence of malnutrition among adult cancer patients receiving chemotherapy treatment was high. Wealth index, food security, appetite loss, presence of diarrhea, and performance status were found to be significant factors for nutritional status. To improve the nutritional status of adult cancer patients, regular nutritional assessment, economic support, improving food security, managing chemotherapy-induced symptoms, such as appetite loss and diarrhea, nutritional education mainly on food preference, energy, and nutrient balance, nutritional intervention, and further longitudinal studies are needed to explore the impact of malnutrition on survival and quality of life in cancer patients.

### 5.1. Recommendation

Governmental and nongovernmental organizations should provide support to cancer patients that have an economic problem to improve food security so that they could not expose to stress and nutritional problems. Health-care providers should consider regular nutritional assessment, manage chemotherapy-induced symptoms, such as appetite loss and diarrhea, and give nutritional education mainly on food preference, energy, and nutrient balance. Lastly, further prospective studies are needed to explore the impact of malnutrition on survival and quality of life among cancer patients in developing countries.

### 5.2. Implication

This study identifies key factors that affect the nutritional status of cancer patients in developing countries. Identification of those predictor factors would permit the government to design strategies to foster the nutritional status of cancer patients. The study finding implies the predictors of malnutrition in cancer patients were low socioeconomic status and symptom burden. Hence, this finding emphasizes to give economic support to less wealthy cancer patients and better management of cancer-related symptoms should be included to improve the nutritional status of patients.

## Figures and Tables

**Table 1:** Socio-demographic characteristics of adult cancer patients under chemotherapy treatment at HUCSH, Hawassa, Southern Ethiopia, 2021.

Variables	Category	Frequency	Percent
Age	18–40	102	31.5
	41–49	85	26.2
	50–59	97	29.9
	≥60	40	12.3

Sex	Female	223	68.8
	Male	101	31.2

Residences	Rural	215	66.4
	Urban	109	33.9

Ethnicity	Sidama	209	64.5
	Amhara	46	14.2
	Oromo	46	14.2
	Gurage	23	7.1
	Other		

Religion	Orthodox	18	5.6
	Muslim	23	7.1
	Protestant	256	79

Marital status	Married	231	71.3
	Single	22	6.8
	Divorced	38	11.7
	Widowed	33	10.2

Educational status	No formal education	176	54.3
	Primary	89	27.5
	Secondary	30	9.3
	College and above	29	9.0

Occupational status	Housewife	125	37.7
	Farmer	90	27.4
	Merchant	50	15.5
	Government employee	30	9.4
	Unemployed	16	4.9
	Other^*∗*^	13	4.0

Wealth index	Lowest	65	20.1
	Second	109	33.6
	Middle	48	14.8
	Fourth	64	19.8
	Highest	38	11.7

**Table 2:** Clinical characteristics of adult cancer patients undertaking chemotherapy treatment at HUCSH, Hawassa, Southern Ethiopia, 2021.

Variables	Category	Frequency	Percent
Cancer type	Breast cancer	79	25.5
	GI cancer^*∗*^a	74	23.9
	Gynecological cancer^*∗*^b	68	21.9
	Hematological cancer	57	18.4
	Lung cancer	17	5.5
	Prostate cancer	15	4.8

Duration of illness	<12 months	218	70.3
	12–24 months	29	9.4
	25–36 months	22	7.1
	37–48 months	16	5.2
	49–60 months	14	4.5
	>60 months	11	3.5

Cycle of chemotherapy	Two	63	20.3
	Three	62	20
	Four	89	28.7
	Five and above	96	31

Stage of cancer	Stage I	18	5.8
	Stage II	68	21.9
	Stage III	131	42.3
	Stage IV	93	30

Comorbidity	No comorbidity	239	77.1
	Hypertension	39	12.6
	DM	16	5.2
	Cardiac diseases	9	2.9
	Other^*∗*^c	7	2.3

WHO performance status	<2	97	29.9
	≥2	227	70.1

Anxiety	Normal	192	59.3
	Border line	83	25.6
	Case	49	15.1

Depression	Normal	164	50.6
	Border line	90	27.8
	Case	70	21.6

^*∗*^a, GI cancer: esophageal, gastric, and colorectal cancers. ^*∗*^b, gynecologic cancer: ovarian and cervical cancers.

**Table 3:** Nutritional status of adult cancer patients undertaking chemotherapy treatment at HUCSH, Hawassa, Southern Ethiopia.

Nutritional status		Frequency	Percent
PG-SGA score	Well nourished	168	51.9
	Moderately malnourished	118	36.4
	Severely malnourished	38	11.7

**Table 4:** Bivariable and multivariable logistic regression of determinant factors for nutritional status among adult cancer patients under chemotherapy at HUCSH, Hawassa, Southern Ethiopia.

Variables	Category	Nutritional status		COR (95 CI)	AOR (95 CI)	*P* value
		Undernutrition	Normal			
Wealth index	Lowest	43	10	0.1 (0.02–0.16)	0.06 (0.02–0.2)	<0.001^*∗∗*^
	Second	52	52	0.2 (0.10–0.57)	0.3 (0.1–0.75)	
	Middle	24	31	0.3 (0.12–0.8)	0.3 (0.09–0.99)	
	Fourth	29	42	0.3 (0.14–0.87)	0.4 (0.15–1.29)	
	Highest	8	33	1	1	

Food security	Insecure	81	20	8.0 (4.553–14.030)	0.1 (0.05–0.24)	<0.001^*∗∗*^
	Secure	75	148	1	1	

Appetite lose	Yes	132	79	0.3 (0.178–0.476)	0.2 (0.11–0.44)	<0.001^*∗∗*^
	No	124	89	1	1	

Diarrhea	Yes	89	71	1	1	0.005^*∗∗*^
	No	67	97	1.8 (1.17–2.82)	2.6 (1.34–5.00)	0.01^*∗*^

WHO-PS	<2	65	32	0.3 (0.2–0.543)	0.4 (0.18–0.79)
	> 2	91	136	1	1	

^*∗∗*^Significant level at *p* < 0.01, ^*∗*^significant level at *p* < 0.05.

## Data Availability

The datasets analyzed during the current study are available from the corresponding author upon reasonable request.

## Abbreviations

AOR: Adjusted odds ratio
CI: Confidence interval
COR: Crude 275 odds ratio
HUCSH: Hawassa University Comprehensive Specialized Hospital
IL: Illinois
PCA: Principal component analysis
PG-SGA: Patient-generated subjective global assessment
SGA: Subjective global assessment
SPSS: Statistical package for social science
USA: United States of America
WHO-PS: World Health Organization Performance Status.
